# Impact of *GBA1* variants on long-term clinical progression and mortality in incident Parkinson’s disease

**DOI:** 10.1136/jnnp-2020-322857

**Published:** 2020-04-17

**Authors:** Thomas B Stoker, Marta Camacho, Sophie Winder-Rhodes, Ganqiang Liu, Clemens R Scherzer, Thomas Foltynie, Jonathan Evans, David P Breen, Roger A Barker, Caroline H Williams-Gray

**Affiliations:** 1 John van Geest Centre for Brain Repair, Department of Clinical Neurosciences, University of Cambridge, Cambridge, Cambridgeshire, UK; 2 Wellcome Trust Medical Research Council - Cambridge Stem Cell Institute, Cambridge, UK; 3 School of Medicine, Sun Yat-Sen University, Guangzhou, Guangdong, China; 4 Advanced Center for Parkinson's Disease Research, Brigham and Women's Hospital, Boston, Massachusetts, USA; 5 Precision Neurology Program, Brigham and Women's Hospital, Boston, Massachusetts, USA; 6 Department of Clinical and Movement Neurosciences, UCL Institute of Neurology, London, UK; 7 Department of Neurology, Nottingham University Hospitals NHS Trust, Nottingham, UK; 8 Centre for Clinical Brain Sciences, University of Edinburgh, Edinburgh, UK; 9 Anne Rowling Regenerative Neurology Clinic, University of Edinburgh, Edinburgh, UK; 10 Usher Institute of Population Health Sciences and Informatics, University of Edinburgh, Edinburgh, UK

## Abstract

**Introduction:**

Variants in the *GBA1* gene have been identified as a common risk factor for Parkinson’s disease (PD). In addition to pathogenic mutations (those associated with Gaucher disease), a number of ‘non-pathogenic’ variants also occur at increased frequency in PD. Previous studies have reported that pathogenic variants adversely affect the clinical course of PD. The role of ‘non-pathogenic’ *GBA1* variants on PD course is less clear. In this study, we report the effect of *GBA1* variants in incident PD patients with long-term follow-up.

**Methods:**

The study population consisted of patients in the Cambridgeshire Incidence of Parkinson’s disease from General Practice to Neurologist and Parkinsonism: Incidence, Cognition and Non-motor heterogeneity in Cambridgeshire cohorts. Patients were grouped into non-carriers, carriers of ‘non-pathogenic’ *GBA1* variants and carriers of pathogenic *GBA1* mutations. Survival analyses for time to development of dementia, postural instability and death were carried out. Cox regression analysis controlling for potential confounders were used to determine the impact of *GBA1* variants on these outcome measures.

**Results:**

*GBA1* variants were identified in 14.4% of patients. Pathogenic and ‘non-pathogenic’ *GBA1* variants were associated with the accelerated development of dementia and a more aggressive motor course. Pathogenic *GBA1* variants were associated with earlier mortality in comparison with non-carriers, independent of the development of dementia.

**Discussion:**

*GBA1* variants, including those not associated with Gaucher disease, are common in PD and result in a more aggressive disease course.

## Introduction

Heterozygous mutations in the *GBA1* gene have been identified as a common risk factor for Parkinson’s disease (PD), being found in approximately 5% of patients.^1–4^ Biallelic pathogenic mutations result in the lysosomal storage disorder, Gaucher disease (GD).^5^ In addition to the known pathogenic mutations associated with GD, there are a number of ‘non-pathogenic’ *GBA1* variants that are not associated with GD but that are found at increased frequency in patients with PD, including the common E326K and T369M variants.^6–9^ Carrying a pathogenic *GBA1* mutation conveys an earlier age of onset^10 11^ and adversely affects the clinical course of PD, manifested by an increased risk of cognitive decline and dementia^1 10 12–18^ and increased rate of motor progression.^1 10 12 19 20^ However, some studies have found no effect on motor progression in *GBA1* mutation-associated PD.^14 15 17^ Few studies have commented on the effect of *GBA1* mutations on mortality in PD, though some have suggested that survival time is reduced in *GBA1* mutation carriers.^10 16^ The clinical significance of carrying a ‘non-pathogenic’ *GBA1* variant is disputed^14 21^ though the E326K variant in particular has been associated with an adverse clinical course in PD in some studies.^20 22^

We previously reported on the epidemiology of *GBA1* mutations in incident PD.^1^ Here we present long-term follow-up data of incident PD cases carrying pathogenic and ‘non-pathogenic’ variants in the *GBA1* gene from two community-based cohorts: the ‘Cambridgeshire Incidence of Parkinson’s disease from General Practice to Neurologist’ (CamPaIGN) and ‘Parkinsonism: Incidence, Cognition and Non-motor heterogeneity in Cambridgeshire’ (PICNICS) cohorts. Advantages of this study over some previously published studies include the long duration of follow-up, along with the fact that both cohorts consisted of community-based patients with newly diagnosed PD.

## Methods

### Patients

The study population consisted of patients in the CamPaIGN and PICNICS cohorts—both community-based incident PD cohorts table 1 and online supplementary table 3).^23^ PD diagnosis was determined by the UK Parkinson’s Disease Society Brain Bank Criteria. The CamPaIGN cohort was recruited between 2000 and 2002, and subjects were followed up at 2 yearly intervals for up to 18 years. The PICNICS cohort was recruited between 2006 and 2013, and subjects have been followed up at 18 monthly intervals, so far for up to 11.4 years. Inclusion and exclusion criteria for these cohorts are summarised in online supplementary table 1.Written informed consent was obtained from all subjects.

10.1136/jnnp-2020-322857.supp1 Supplementary data

**Table 1:** Patient demographics

	Non-carriers	All *GBA1* variants	P value	*GBA1* non-pathogenic variant carriers	Pathogenic *GBA1* mutation carriers	P value
Number	214	48	–	31	17	–
Sex M:F	127:87	32:16	0.348	22:9	10:7	0.459
Age at diagnosis	69.2 (9.8)	68.3 (8.9)	0.569	68.7 (10.1)	67.5 (6.3)	0.785
Years of follow-up	6.9 (4.2)	6.1 (3.4)	0.255	6.8 (3.6)	6.2 (2.8)	0.791
Years in education	11.9 (3.1)	11.3 (2.5)	0.253	11.3 (2.5)	11.5 (2.7)	0.510
Total UPDRS at baseline	51.8 (19.6)	54.2 (16.8)	0.431	56.2 (18.3)	50.0 (15.2)	0.452
Motor UPDRS at baseline	31.6 (12.5)	35.1 (12.0)	0.072	35.7 (12.1)	35.1 (13.2)	0.168
Hoehn and Yahr at baseline	1.7 (0.7)	2.0 (0.8)	**0.02**	2.0 (0.74)	2.0 (0.8)	0.067
PIGD score at baseline	0.75 (0.55)	0.85 (0.47)	0.225	0.86 (0.52)	0.79 (0.34)	0.563
Tremor score at baseline	0.59 0.41)	0.61 (0.41)	0.744	0.75 (0.42)	0.51 (0.35)	0.119
Baseline levodopa equivalent dose	185.9 mg (238.7)	204.4 mg (241.1)	0.594	166.1 mg (214.1)	274.1 mg (277.0)	0.290
MMSE at baseline	28.5 (1.4)	28.2 (1.9)	0.274	27.9 (2.1)	28.6 (1.4)	0.150
BDI at baseline	7.3 (5.3)	6.8 (5.0)	0.629	7.8 (5.6)	4.9 (2.9)	0.180
NART at baseline	116.1 (61.9)	111.3 (11.3)	0.609	109.7 (10.7)	113.2 (12.0)	0.873
PDQ-39 at baseline	24.7 (19.8)	26.4 (19.8)	0.602	32.5 (22.3)	17.5 (9.2)	0.057

SDs shown in brackets.

P values are shown for comparison between non-carriers and all variant carriers as determined by independent samples t-tests and χ^2^ tests, and for comparison between non-carriers, ‘non-pathogenic’ variant carriers and pathogenic variant carriers, as determined by one-way analysis of variance and χ^2^ tests. Statistically significant results highlighted in bold.

BDI, Beck Depression Inventory; F, female; M, male; MMSE, Mini-Mental State Examination; NART, National Adult Reading Test; PDQ-39, Parkinson’s Disease Questionnaire 39; PIGD, postural instability and gait disability; UPDRS, Unified Parkinson’s Disease Rating Scale.

Motor, cognitive and neuropsychiatric assessments were completed at each time point including the Unified Parkinson’s Disease Rating Scale (UPDRS) in the CamPaIGN cohort, Movement Disorders Society UPDRS (MDS-UPDRS) in the PICNICS cohort, Mini-Mental State Examination (MMSE), Hoehn and Yahr (HY) stage, levodopa equivalent dose, Beck Depression Inventory, National Adult Reading Test and Parkinson’s Disease Questionnaire-39. For the CamPaIGN cohort, estimated MDS-UPDRS scores were calculated as described previously.^24^ Postural instability and gait disability (PIGD) and tremor scores were calculated for the CamPaIGN cohort as described here.^25^ We adapted this method to allow calculation of similar motor phenotypes in the PICNICS cohort, using the mean value of the UPDRS-MDS scores for questions 2.10 (tremor history), 3.15 (postural tremor), 3.16 (kinetic tremor) and 3.17 (rest tremor) for tremor score, and questions 2.12 (walking and balance history), 2.13 (freezing history), 3.10 (gait examination) and 3.13 (posture examination) for PIGD score. *GBA1* variants were identified through sequencing of the *GBA1* gene targeted at exons 1 to 11 in 250 patients, as described previously.^1^ A further 127 patients underwent targeted screening using an Illumina Multi-Ethnic Genotyping Array (MEGA) ChIP for specific *GBA1* variants (online supplementary figure 1).^26^

### Survival and regression analyses

Subjects were grouped into non-carriers and carriers of any *GBA1* variant (*GBA1*-PD). The *GBA1*-PD group was further stratified into carriers of pathogenic mutations and carriers of ‘non-pathogenic’ variants, as indicated below. Hereafter, variants referred to as ‘pathogenic’ are those associated with the development of GD in the homozygous or compound heterozygous state, and variants referred to as ‘non-pathogenic’ are those that are not associated with the development of GD. All patients in whom sequencing had been undertaken were included in survival analyses, as well as additional patients in whom any *GBA1* variant was detected on the MEGA ChIP array. Patients in which only MEGA ChIP screening was performed that were not found to carry a *GBA1* variant were excluded, given the possibility that they carried an undetected *GBA1* variant that had not been screened for. For subgroup analysis, subjects that only had genotyping with the Mega ChIP array in which no *GBA1* variant was identified, or in which a ‘non-pathogenic’ variant was identified (n=6), were excluded to ensure that there no patients with missed pathogenic mutations were included in the non-carrier or ‘non-pathogenic’ variant carrier groups.

Time to mortality, postural instability and dementia were selected as outcome measures, as these represent important clinical milestones in the progression of PD. Kaplan-Meier curves and Cox Regression analyses controlling for potential confounders as detailed below were used for comparison between the groups. Development of postural instability was defined as time to reach HY stage three (HY3) or PIGD score of 2 or more, and dementia was defined as a MMSE score of 24 or less, with fulfilment of *Diagnostic and Statistical Manual of Mental Disorders IV* criteria (functional impairment (as defined by Schwab and England score of 70% or less) with cognitive deficits in at least two domains and exclusion of other causes of cognitive dysfunction). The CamPaIGN and PICNICS cohort studies predated establishment of the Movement Disorder Society PD dementia criteria. Patients were only considered to have reached these outcomes if these criteria were fulfilled at all subsequent visits.

### Statistical analysis

All statistical tests were performed on SPSS V.25 software. Independent samples t-tests were used to compare between non-carriers and carriers of all *GBA1* variants. Levene’s test was first performed to ensure that equality of variance could be assumed. One-way analysis of variance tests were used for comparison between non-carriers and carriers of ‘non-pathogenic’ or pathogenic *GBA1* variants. χ^2^ tests were used to compare sex distribution. For survival analysis, log-rank values are reported, with comparison with the non-carrier group.

## Results

### Incidence and spectrum of GBA1 abnormalities

*GBA1* variants were present in 36 out of the 250 patients in which sequencing was performed (14.4%). Pathogenic mutations were present in 11 of these patients (4.4 %), which included N370S (n=3), L444P (n=3), R463C (n=1), G10S (n=1), N426K (n=1), R48W (n=1) and R257Q (n=1). Isolated ‘non-pathogenic’ variants were present in 25 of these patients (10.0 %). These included c.762 18T>A (n=8), E326K (n=7), T369M (n=7), E388K (n=1), L119L (n=1) and c.589 86A>G) (n=1). The carrier of the pathogenic R48W variant carried a concomitant E326K variant, and the carrier of the pathogenic N426K variant carried a concomitant c.762 18T>A variant. These were both included in the pathogenic variant group for subgroup analyses. Variants were identified in 12 of the 127 patients (9.4%) in which MEGA ChIP analysis was performed. These included the pathogenic N370S (n=4), R463C (n=1) and G10S (n=1) variants and the ‘non-pathogenic’ T369M (n=3), E326K (n=2) and E388K (n=1) variants.

After excluding those patients in which only MEGA ChIP genotyping was performed that were not identified to carry a *GBA1* variant, these combined cohorts contained 214 non-carriers and 48 carriers of *GBA1* variants. Mean follow-up times were 8.0 years (maximum 18.0 years) for the CamPaIGN cohort (n=115) and 5.8 years (maximum 11.4 years) for the PICNICS cohort (n=147).

### Cognitive decline and risk of dementia

**Figure 1:**
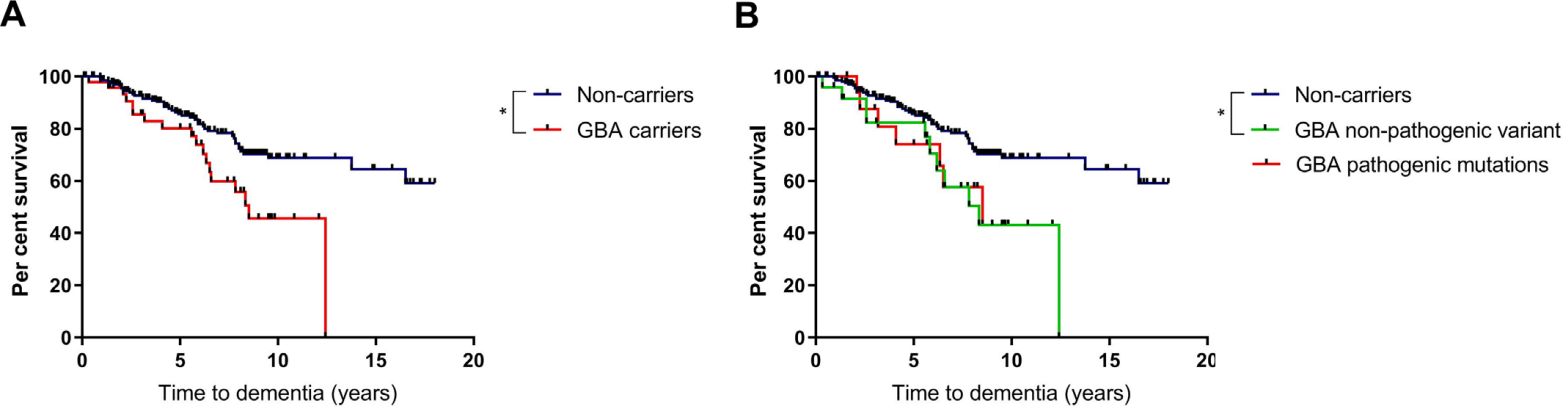
Effect of *GBA1* variants on development of dementia in PD. (A) Survival analysis for time to dementia in carriers of any *GBA1* variant compared with non-carriers. (B) Subgroup survival analysis for time to dementia in carriers of pathogenic *GBA1* mutations, ‘non-pathogenic’ variants and non-carriers. Statistical significance as determined by log-rank tests indicated by asterisk. PD, Parkinson’s disease.

Baseline cognitive function did not differ between variant carriers and non-carriers, with no significant differences in MMSE scores at initial assessment (table 1). Carriers of any *GBA1* variant had an increased risk of progression to dementia compared with non-carriers (table 2 and figure 1). Cox regression analysis controlling for age at diagnosis, sex and baseline MMSE score revealed that development of dementia was significantly more likely in carriers of any *GBA1* variant, compared with non-carriers, with an HR of 2.1 (95% CI 1.2 to 3.9, p=0.013). At the 10-year time point, fewer than half of the variant carriers remained dementia free, in comparison with 68.4% of non-carriers. At 15 years, 64.2% of surviving non-carriers remained dementia-free. All patients in the *GBA1* carrier group had developed dementia or died prior to the 15-year time point. The estimated mean time to dementia was approximately 5 years earlier in variant carriers, compared with non-carriers (table 2).

**Table 2:** Time to dementia

	Non-carriers	All *GBA1* variants	Non-pathogenic variants	Pathogenic mutations
Number	214	48	25	17
Estimated mean time to dementia (years)	13.7 (12.6 to 14.7)	8.6 (7.2 to 10.0)	8.3 (6.4 to 10.2)	8.3 (6.2 to 10.3)
Estimated median time to dementia (years)	*	8.5 (6.9 to 10.1)	8.3 (5.4 to 11.3)	8.5 (4.1 to 12.9)
5-year survival without dementia (%)	85.3 (SE 2.7)	80.2 (SE 6.3)	82.3 (SE 8.0)	74.0 (SE 11.2)
10-year survival without dementia (%)	68.4 (SE 4.3)	45.7 (SE 9.8)	43.2 (SE 12.5)	43.2 (SE 16.0)
15-year survival without dementia (%)	64.2 (SE 5.8)	0	0	0
Log-rank p value	–	**0.013**	**0.014**	0.109
HR	–	**2.1 (1.2 to 3.9; p=0.013**)	1.8 (0.9 to 3.8; p=0.110)	**3.6 (1.6 to 8.3; p=0.003**)

Long-term follow-up data for progression to dementia in PD patients without *GBA1* variants compared with those carrying any *GBA1* variant, a pathogenic *GBA1* mutation or a *GBA1* ‘non-pathogenic’ variant.

HR determined by Cox regression analysis controlling for age at diagnosis, sex and baseline MMSE score. Ninety-five per cent CI for HR and estimated mean and median shown in brackets. Log-rank p values shown for comparison between the GBA1 groups and the non-carrier group. Statistically significant results highlighted in bold.

*Fewer than half reached outcome

MMSE, Mini-Mental State Examination.

Subclassification of the variant carrier group into those with pathogenic mutations and those with ‘non-pathogenic’ variants revealed a significantly increased risk of progression to dementia in carriers of pathogenic variants, with an HR of 3.6 (95% CI 1.5 to 8.3, p=0.003). In carriers of ‘non-pathogenic’ variants, there was a non-significant trend towards increased risk of dementia (HR 1.8 (95% CI 0.9 to 3.8, p=0.11).

### Motor progression

**Figure 2:**
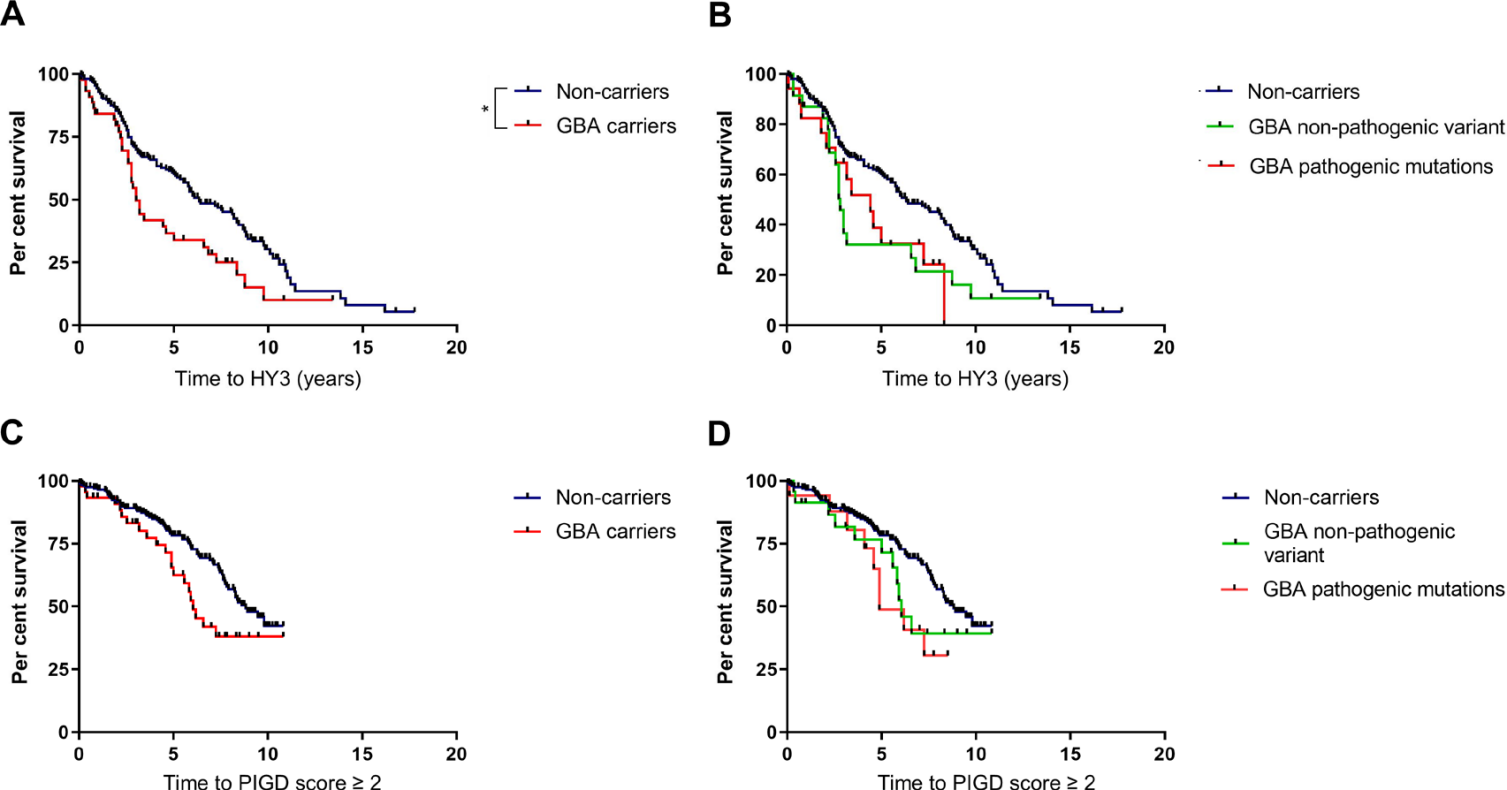
Effect of *GBA1* variants on development of postural instability in PD. (A and C) Survival analysis for time to HY3 and PIGD score of 2 or more, respectively, in carriers of any *GBA1* variant compared with non-carriers. (B and D) Subgroup survival analysis for time to HY3 and PIGD score of 2 or more, respectively, in carriers of pathogenic *GBA1* mutations, ‘non-pathogenic’ variants and non-carriers. Statistical significance as determined by log-rank tests indicated by asterisk. HY3, Hoehn and Yahr stage three; PIGD, postural instability and gait disability.

The baseline HY stage was lower in non-carriers compared with carriers (table 1), though there were no significant differences between the subgroups. There were no differences in baseline UPDRS motor score, PIGD score or levodopa equivalent dose between any of the groups. The risk of progression to postural instability was increased in the *GBA1*-PD group in comparison with the non-carrier group (table 3 and figure 2). At 5 years from diagnosis, 67.5% of the *GBA1*-PD group had reached HY3, compared with 43% of non-carriers. After controlling for potential confounders (age at diagnosis, sex, baseline HY score, baseline UPDRS motor score and baseline PIGD score), the risk of progression to HY3 was significantly greater in both carriers of ‘non-pathogenic’ variants and pathogenic mutations, with an HR of 1.7 (95% CI 1.0 to 2.9, p=0.035) and 1.9 (95% CI 1.0 to 3.4, p=0.036), respectively (figure 2 and table 3).

**Table 3:** Time to HY3

	Non-carriers	All *GBA1* variants	Non-pathogenic variants	Pathogenic mutations
Number	213	48	25	17
Estimated mean time to HY3 (years)	6.8 (5.9 to 7.6)	4.7 (3.4 to 5.9)	4.6 (3.0 to 6.3)	4.4 (3.0 to 5.9)
Estimated median time to HY3 (years)	6.0 (4.6 to 7.4)	3.0 (2.5 to 3.5)	2.8 (2.5 to 3.1)	4.4 (2.6 to 6.2)
5-year survival without postural instability (%)	57.0 (SE 3.6)	32.5 (SE 7.3)	32.0 (SE 10.0)	32.4(SE 11.8)
10-year survival without postural instability (%)	28.7 (SE 4.1)	9.6 (SE 5.9)	10.7 (SE 7.0)	0
15-year survival without postural instability (%)	7.7 (SE 3.9)	9.6 (SE 5.9)	10.7 (SE 7.0)	0
Log-rank p value	–	**0.013**	0.074	0.095
HR	–	**1.7 (1.2 to 2.6; p=0.006**)	**1.7 (1.0 to 2.9; p=0.035**)	**1.9 (1.0 to 3.4; p=0.036**)

Long-term follow-up data for progression to postural instability in PD patients without *GBA1* variants compared with those carrying any *GBA1* variant, a pathogenic *GBA1* mutation or a *GBA1* ‘non-pathogenic’ variant.

HR determined by Cox regression analysis controlling for age at diagnosis, sex, baseline HY score, baseline UPDRS motor score and baseline PIGD score. Ninety-five per cent CI for HR and estimated mean and median shown in brackets. Log-rank p values shown for comparison between the GBA1 groups and the non-carrier group. Statistically significant results highlighted in bold.

HY3, Hoehn and Yahr stage three; PIGD, postural instability and gait disability; UPDRS, Unified Parkinson’s Disease Rating Scale.

Time to PIGD score of 2 or more was also determined as a measure of motor progression. Pathogenic variants were associated with an increased risk of progression to PIGD score of 2 or more, with a HR of 2.8 (95% CI 1.4 to 5.7, p=0.005). There was no significant increased risk of progression to PIGD score of two or more in carriers of ‘non-pathogenic’ variants (table 4).

**Table 4:** Time to PIGD score of 2 or more

	Non-carriers	All *GBA1* variants	Non-pathogenic variants	Pathogenic mutations
Number	214	48	25	17
Estimated mean time to PIGD ≥2 (years)	7.7 (7.2 to 8.3)	6.8 (5.6 to 7.9)	6.9 (5.3 to 8.5)	5.7 (4.4 to 7.0)
Estimated median time to PIGD ≥2 (years)	8.6 (7.4 to 9.9)	6.0 (5.1 to 7.0)	6.0 (5.1 to 7.0)	4.9 (3.1 to 6.6)
5-year survival to PIGD ≥2 (%)	76.1 (SE 3.2)	62.6 (SE 8.1)	71.5 (SE 9.9)	48.8 (SE 13.9)
10-year survival to PIGD ≥2 (%)	40.9 (SE 5.6)	37.9 (SE 8.8)	39.0 (SE 12.0)	30.5 (SE 13.5)
Log-rank p value	–	0.094	0.335	0.066
HR	–	**1.9 (1.1 to 3.2; p=0.015**)	1.6 (0.8 to 3.1; p=0.178)	**2.8 (1.4 to 5.7; p=0.005**)

Long-term follow-up data for progression to postural instability in PD patients without *GBA1* variants compared with those carrying any *GBA1* variant, a pathogenic *GBA1* mutation or a *GBA1* ‘non-pathogenic’ variant.

HR determined by Cox regression analysis controlling for age at diagnosis, sex, baseline HY score, baseline UPDRS motor score and baseline PIGD score. Ninety-five per cent CI for HR and estimated mean and median shown in brackets. Log-rank p values shown for comparison between the GBA1 groups and the non-carrier group. Statistically significant results highlighted in bold.

HY3, Hoehn and Yahr stage three; PIGD, postural instability and gait difficulty score; UPDRS, Unified Parkinson’s Disease Rating Scale.

### Mortality

**Figure 3:**
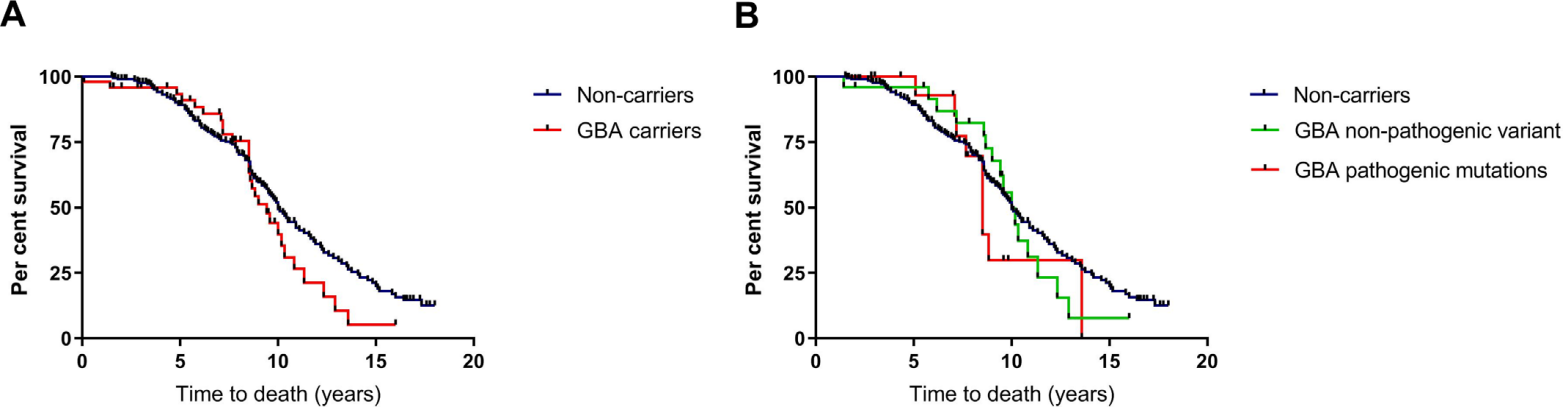
Effect of *GBA1* variants on time to death in PD. (A) Survival analysis for time to death in carriers of any *GBA1* variant compared with non-carriers. (B) Subgroup survival analysis for time to death in carriers of pathogenic *GBA1* mutations, ‘non-pathogenic’ variants and non-carriers. PD, Parkinson’s disease.

Mean time to mortality was approximately 1 year earlier in carriers of any *GBA1* variant compared with non-carriers (table 5 and figure 3). At 15 years from diagnosis, only 5.3% of *GBA1* variant carriers were surviving, compared with 20.1% of non-carriers. When controlling for age and sex, the risk of mortality was increased in carriers of pathogenic mutations in comparison with non-carriers with an HR of 2.1 (95% CI 1.0 to 4.1, p=0.043) but not in those carrying ‘non-pathogenic’ variants (table 5).

**Table 5:** Time to mortality

	Non-carriers	All *GBA1* variants	Non-pathogenic variants	Pathogenic mutations
Number	214	48	25	17
Estimated mean time to death (years)	10.6 (10.0 to 11.3)	9.4 (8.4 to 10.5)	10.0 (8.7 to 11.3)	9.5 (7.8 to 11.3)
Estimated median time to death (years)	10.0 (9.3 to 10.7)	9.4 (8.3 to 10.6)	10.0 (8.9 to 11.1)	8.5 (7.7 to 9.3)
5-year survival (%)	89.2 (SE 2.2)	93.4 (SE 3.7)	96.0 (SE 3.9)	100.0
10-year survival (%)	49.6 (SE 3.9)	39.8 (SE 8.8)	49.7 (SE 11.6)	29.8 (SE 14.1)
15-year survival (%)	20.1 (SE 3.9)	5.3 (SE 5.0)	7.8 (SE 7.3)	0
Log-rank p value	–	0.169	0.639	0.275
HR	–	1.5 (1.0 to 2.3; p=0.061)	1.2 (0.7 to 2.0; p=0.567)	**2.1 (1.0 to 4.1; p=0.043**)

Long-term follow-up data for progression to time to mortality in PD patients without GBA1 variants compared with those carrying any *GBA1* variant, a pathogenic *GBA1* mutation or a *GBA1* ‘non-pathogenic’ variant.

HR determined by Cox regression analysis controlling for age at diagnosis and sex. Ninety-five per cent CI for HR and estimated mean and median shown in brackets. Log-rank p values shown for comparison between the GBA1 groups and the non-carrier group. Statistically significant results highlighted in bold.

PD, Parkinson’s disease.

To explore whether the increase in mortality associated with pathogenic *GBA1* variants was attributable to the development of dementia, the dementia outcome was added into the Cox regression model, along with age and sex. The increased risk of mortality associated with carrying any *GBA1* variant persisted after controlling for dementia status (HR 2.0 (95% CI 1.0 to 4.1, p=0.046)), suggesting that the relationship was independent of the development of dementia. Causes of death in each group are shown in online supplementary table 2.

## Discussion

In this study, we have described the clinical course of incident *GBA1*-PD for up to 18 years from the time of diagnosis, which is a longer follow-up period than any previous study. We have demonstrated that *GBA1* variants confer an increased risk of dementia, faster motor progression and mortality in PD, in keeping with previous reports. Importantly, in addition to pathogenic *GBA1* mutations, variants generally considered to be ‘non-pathogenic’ were found to adversely affect disease course in *GBA1*-PD.

By studying patients with incident PD for up to 18 years, this study has uniquely described the natural history of *GBA1*-PD. Most prior longitudinal studies of *GBA1*-PD have employed prevalent cohorts in which variation in disease duration may confound the results, and many other studies have used clinic-based cohorts that may be disproportionately represented by atypical or severe cases. The two cohorts analysed in this study were community based and thus more representative of the general PD population. Another strength of this study was the inclusion of patients with *GBA1* variants that do not cause GD, which occur at increased incidence in PD. These variants were termed ‘non-pathogenic’ to differentiate them from variants that cause GD, though taking into account results from this and previous studies, they should not be considered to be non-deleterious.

Of the patients that had undergone genetic sequencing, 14% carried a *GBA1* variant, with 4.4% carrying a pathogenic mutation^2–4 27^ previously associated with GD. The frequency of pathogenic mutations was similar to that seen in previous studies. The prevalence of any *GBA1* variant was higher than that seen in other studies, probably due to the fact that we included non-coding variants such as c.762 18T>A, which has been reported as a potential risk factor for PD, though its importance is not clear.^28^ The most common variants were c.762 18T>A, E326K and T369M, which accounted for almost two-thirds of all variants. Carriers of these variants are a particularly interesting group to study, as their clinical significance is much less clear than that of so-called ‘pathogenic’ *GBA1* mutations.

We found that *GBA1* variants were generally associated with a more aggressive disease course. Diagnosis of dementia and development of postural instability were chosen as outcome measures, since these represent important milestones in the clinical progression of PD.^29^ Consistent with previous studies, carriers of pathogenic mutations had an increased risk of reaching both of these milestones. Carriers of any *GBA1* variant had a projected mean time to dementia that was approximately 5 years earlier than in non-carriers, while the estimated mean time to development of postural instability was approximately 2 years earlier than that seen in non-carriers. Carriers of pathogenic mutations had a 3.6-fold greater risk of development of dementia than in non-carriers and a 1.9-fold greater risk of progression to HY3. Importantly, carriers of variants not associated with GD conveyed a similar risk of development of postural instability in comparison with non-carriers, with a trend towards greater risk of dementia. These variants have previously been reported to have little effect on clinical course in PD,^14^ though it is possible that such effects would be missed due to short follow-up times and heterogeneous populations. Carriers of the common E326K variant have been shown to have an increased risk of dementia and motor progression in comparison with non-carriers in one US-based study.^20^ However, in a large meta-analysis of *GBA1* variants in PD, there was no association between development of PD and the E326K variant.^4^ This study population was enriched for individuals from Jewish, Portuguese and Asian populations, in which the E326K accounts for a lower proportion of *GBA1* variants than in other populations, which may account for the lack of association in this analysis.^3 30^

We also found a significantly increased risk of mortality over the study period in carriers of pathogenic *GBA1* variants, in which the risk of death was approximately twofold greater than that in non-carriers, with a mean time to mortality approximately 1 year earlier. This observation was not attributable to the increased incidence of dementia in *GBA1* variant carriers, since the effect persisted after controlling for dementia status. Other factors (such as immobility, adverse drug effects and non-motor features) may contribute to the observed increase in mortality in *GBA1*-PD. ‘Non-pathogenic’ variants were not associated with an increased risk of mortality, though the time to mortality was non-significantly reduced in this group. The impact of *GBA1* variants on PD progression may lie on a spectrum, with pathogenic mutations conveying a greater biological effect than ‘non-pathogenic’ variants. As such, our study may have been underpowered to detect a small reduction in time to mortality associated with ‘non-pathogenic’ variants, and it would be interesting to explore this in a larger cohort.

Age of disease onset has previously been reported to be reduced in PD associated with *GBA1* mutations.^10 13 27 31^ In this study, age of onset did not differ based on *GBA1* status. The mean age of onset in both carriers of pathogenic mutations and carriers of ‘non-pathogenic’ variants in *GBA1* was approximately 2 years earlier than in non-carriers, and it may be that this study was underpowered to detect such a difference in age at onset.

The observation that ‘non-pathogenic’ *GBA1* variants adversely affect the prognosis of PD patients is important. This significantly increases the proportion of patients with PD in which *GBA1* abnormalities play a potentially pathogenic role, in comparison with considering GD-causative mutations as the only variants that alter PD course. Given that *GBA1* mutations are associated with the specific pathology of lysosome–autophagy system dysfunction,^32^ it is possible that the optimal treatment approach in this group of patients will involve drugs that are able to restore activity of this system. As such treatments emerge, it may become important to screen patients with PD for variants in the *GBA1* gene, so that precision medicine can be applied, and our data suggest that it will be important to screen for a much broader range of variants than those previously identified as ‘pathogenic’.

The ‘pathogenic’ mutations associated with GD result in significant reduction in glucocerebrosidase enzyme activity, which underlies its clinical manifestations.^5 33^ However, polymorphisms such as E326K result in a relatively minimal reduction in enzyme activity,^22^ and the fact that these predispose to PD without being associated with GD, supports the concept that the pathogenic mechanisms of *GBA1*-PD are distinct from those of GD. This means that putative treatments for *GBA1-*PD may need to correct glucocerebrosidase folding and structure, or restore activity of dysfunctional pathways (such as the lysosome-autophagy system), rather than simply restoring glucocerebrosidase enzyme activity.

We acknowledge that there are a number of limitations with our study. While we have controlled for relevant potential confounders in our survival analyses, data regarding prodromal features of PD that may influence prognosis (eg, rapid eye movement sleep-behaviour disorder) were not available. We have reported on the time from diagnosis, rather than time from symptom onset, to the development of clinical milestones, which may not truly reflect disease duration. However, the time of symptom onset is difficult to determine accurately, since many prodromal features may not be attributed to PD, which would potentially introduce bias.

These cohorts predated the development of the MDS criteria for PD dementia. Cognitive status was measured in these cohorts with MMSE, though other cognitive examinations are now favoured. In order to maximise the accuracy of dementia diagnosis in these cohorts, we employed strict criteria that reflect the MDS criteria for PD dementia, including a requirement for irreversible deficits in multiple cognitive domains accompanied by functional impairment. Though we included baseline MMSE as a covariate in our Cox regression model for time to dementia, it is not known how many patients had mild cognitive impairment at baseline, which may be expected to influence prognosis. Because assessments were performed every 2 years for the CamPaIGN cohort and every 18 months for the PICNICS cohort, it was not possible to determine the exact time of the onset of dementia or progression to HY3 or PIGD score of 2 or more, which was determined as the midpoint between the assessment at which the patient had reached the outcome measure and the prior assessment.

Another limitation of our study is the relatively limited sample size. We have attempted to address this by using two homogeneous incident cohorts from the same population base but separated in time.^23^ However, 115 patients were excluded from survival analyses as they had only undergone limited genetic screening, meaning that it was possible that they harboured unidentified, potentially clinically significant *GBA1* variants. The sequencing of *GBA1* conferred major benefits in this study, as a more comprehensive method for identifying both presence of absence of *GBA1* variants. While we acknowledge that complete sequencing reads could not be achieved in all patients (as previously described in Winder-Rhodes *et al* 1 2013), we feel that the use of sequencing data allowed us to assign *GBA1* variant carrier status with maximum possible accuracy. Further strengths of this study are the long duration of follow-up of up to 18 years, and the fact that our cohorts consisted of incident PD patients recruited from the community, allowing follow-up from diagnosis to the development of key clinical milestones in a representative population. As such we have presented the first long-term data on the effect of *GBA1* variants on mortality in an incident PD population and detected significant differences in the natural history of PD associated with ‘non-pathogenic’ *GBA1* variants, which may have been missed in previous studies, probably due to their limited follow-up durations.

In conclusion, long-term follow-up of the CamPaIGN and PICNICS cohorts confirms that *GBA1* mutations adversely affect clinical course. Importantly, carriers of *GBA1* variants that are often considered to be ‘non-pathogenic’ had a significantly worse clinical course than non-carriers. *GBA1* variants therefore contribute to the pathogenesis and clinical features of more than 10% of patients with PD, making them a particularly important genetic subgroup. Since *GBA1* abnormalities appear to be associated with specific pathogenic mechanisms, it may be that the optimal disease-modifying approach differs in this group than in the wider PD population, with important implications for future clinical trials.
