# Supplementary material for: Impact of GBA1 variants on long-term clinical progression and mortality in incident Parkinson’s disease
Source: J Neurol Neurosurg Psychiatry. 2020 Apr 17;91(7):695–702. doi: 10.1136/jnnp-2020-322857 (PMC7361014; doi:10.1136/jnnp-2020-322857)
Supplement: Supplementary data [file jnnp-2020-322857supp001.pdf]

Supplementary Material

	CamPaIGN	PICNICS
**Inclusion criteria**	• New cases with Parkinsonism diagnosed December 1 2000 to December 31 2002<br>• Recruited from community (Cambridgeshire)<br>• Diagnosis validated by a movement disorder specialist using UKPD Brain Bank criteria at baseline assessment (with re-validation at 3.5 years)	• New cases of Parkinsons's disease diagnosed between November 1 2006 and May 8 2013<br>• Recruitment from community, Cambridgeshire<br>• UKPD BB criteria at baseline
**Exclusion criteria**	• MMSE of < 24 at baseline<br>• Failure to meet UKPD Brain Bank criteria at subsequent follow-up<br>• Diagnosis other than idiopathic PD	• Dementia at baseline<br>• Failure to meet UKPD Brain Bank criteria at subsequent follow-up<br>• Diagnosis other than idiopathic PD

Supplementary table 1. Inclusion and exclusion criteria for CamPaIGN and PICNICS cohorts.

	Non-carriers	*GBA1* non-pathogenic variant carriers	Pathogenic *GBA1* mutation carriers
**Infection**	**41 (32.5%)**<br>- Respiratory n=36<br>- UTI n=4<br>- UTI + pneumonia n=1	**5 (26.3%)**<br>- Respiratory n=5	**2 (22.2%)**<br>- Respiratory n=1<br>- Infected fracture n=1
**Parkinson's disease**	**14 (11.1%)**	-	**1 (11.1%)**
**Cardiovascular**	**8 (6.3%)**<br>- Congestive cardiac failure n=3<br>- Myocardial / pericardial fibrosis n=2<br>- HOCM n=1<br>- Cardiac arrest n=1<br>- Ruptured AAA n=1	**2 (10.5%)**<br>- Myocardial infarction n=2	-
**Malignancy**	**5 (4.0%)**<br>- Prostate n=1<br>- Bowel n=1<br>- Renal sarcoma n=1<br>- Lung n=1<br>- Not-specified n=1	-	-
**Old age / frailty**	**4 (3.2%)**	-	-
**Stroke**	**2 (1.6%)**	**1 (5.3%)**	-
**Gastrointestinal**	**2 (1.6%)**<br>- Stercoral bowel perforation n=1<br>- Gastrointestinal haemorrhage n=1	**-**	**1 (11.1%)**<br>- Sigmoid volvulus n=1
**Other**	**5 (4.0%)**<br>- COPD n=2<br>- Liver failure n=1<br>- Subdural haematoma n=1<br>- Suicide n=1	-	-
**Unknown**	**45 (35.7%)**	**11 (57.9%)**	**5 (55.6%)**

Supplementary table 2. Causes of death for non-carriers and carriers of "non-pathogenic" and pathogenic *GBA1* variants with Parkinson's disease. Causes of death were determined through analysis of medical certificates of the cause of death where accessible. Abbreviations: AAA = Abdominal aortic aneurysm; COPD = Chronic obstructive pulmonary disease; HOCM = Hypertrophic obstructive cardiomyopathy; UTI = Urinary tract infection.

	**Non-carriers**	**All *GBA1* variants**	**P-value**	***GBA1* non-pathogenic variant carriers**	**Pathogenic *GBA1* mutation carriers**	**P-value**
**Years from diagnosis to baseline visit (SD)**	0.23 (0.33)	0.28 (0.33)	0.344	0.31 (0.35)	0.24 (0.28)	0.534

Supplementary table 3. Time from diagnosis to first assessment for patients in CamPaIGN and PICNICS cohorts.

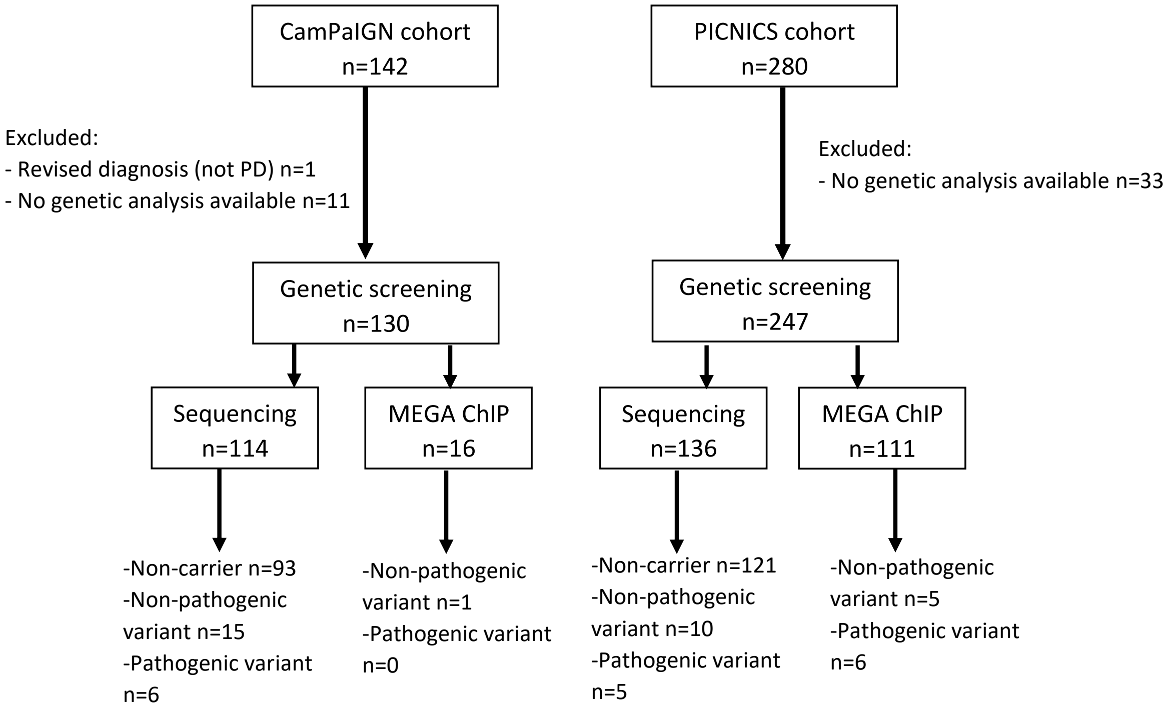

Supplementary figure 1. Genetic analysis in CampaIGN and PICNICS cohorts.
